# Polyphenolic Compounds: Orchestrating Intestinal Microbiota Harmony during Aging

**DOI:** 10.3390/nu16071066

**Published:** 2024-04-05

**Authors:** Quélita Cristina Pereira, Isabela Monique Fortunato, Fabricio de Sousa Oliveira, Marisa Claudia Alvarez, Tanila Wood dos Santos, Marcelo Lima Ribeiro

**Affiliations:** 1 Laboratory of Immunopharmacology and Molecular Biology, Sao Francisco University, Av. Sao Francisco de Assis, 218, Braganca Paulista 12916-900, SP, Brazil; quelita.pereira@mail.usf.edu.br (Q.C.P.); isabela.fortunato@mail.usf.edu.br (I.M.F.); fabricio.sousa@mail.usf.edu.br (F.d.S.O.); marisa.prax@usf.edu.br (M.C.A.); tanila.santos@mail.usf.edu.br (T.W.d.S.); 2 Hematology and Transfusion Medicine Center, University of Campinas/Hemocentro, UNICAMP, Rua Carlos Chagas 480, Campinas 13083-878, SP, Brazil

**Keywords:** aging, dysbiosis, intestinal microbiota, polyphenols, epigenetics

## Abstract

In the aging process, physiological decline occurs, posing a substantial threat to the physical and mental well-being of the elderly and contributing to the onset of age-related diseases. While traditional perspectives considered the maintenance of life as influenced by a myriad of factors, including environmental, genetic, epigenetic, and lifestyle elements such as exercise and diet, the pivotal role of symbiotic microorganisms had been understated. Presently, it is acknowledged that the intestinal microbiota plays a profound role in overall health by signaling to both the central and peripheral nervous systems, as well as other distant organs. Disruption in this bidirectional communication between bacteria and the host results in dysbiosis, fostering the development of various diseases, including neurological disorders, cardiovascular diseases, and cancer. This review aims to delve into the intricate biological mechanisms underpinning dysbiosis associated with aging and the clinical ramifications of such dysregulation. Furthermore, we aspire to explore bioactive compounds endowed with functional properties capable of modulating and restoring balance in this aging-related dysbiotic process through epigenetics alterations.

## 1. Introduction

Aging is characterized as a gradual and continuous process occurring throughout an organism’s lifespan, resulting in physiological alterations. It encompasses a complex interplay of social, cultural, biological, and psychological factors. The global demographic of the elderly is shaped by health conditions, extending beyond mere disease absence to encompass indices of independence and functionality, providing a comprehensive perspective on well-being [1]. The aging process is influenced by a complex interplay of genetic and environmental factors. Over time, these factors contribute to the gradual functional deterioration of cells, tissues, and organs, leading to a decline in physiological integrity. This can potentially increase vulnerability to various pathologies, making aging a significant factor in overall mortality risk [2,3]. The aging process involves characteristics based on three premises: the first involves its manifestation associated with age, the second is characterized by the acceleration of aging, which can be accentuated experimentally, and the third involves the opportunity to decelerate or reverse aging through therapeutic interventions. In this sense, López-Otín and colleagues proposed an expansion related to aging markers, suggesting twelve characteristics of aging that include genomic instability, telomere attrition, epigenetic alterations, loss of proteostasis, deficient macroautophagy, dysregulated nutrient sensing, mitochondrial dysfunction, cellular senescence, stem cell exhaustion, altered intercellular communication, chronic inflammation, and dysbiosis [4].

In the aging process, a progressive decline in physiological function poses a significant threat to the physical and mental well-being of the elderly population, contributing significantly to the onset of age-related diseases. Preventing such maladies, enhancing life quality, and preserving elderly health have become focal points in various research endeavors. Historically, the preservation of life was conventionally attributed to a complex interplay of environmental, genetic, epigenetic factors, and lifestyle components, including physical activity and dietary habits. However, the substantive role played by symbiotic microorganisms in this context was previously underappreciated [5,6].

The balance between commensal and pathogenic bacterial populations is crucial for the integrity of the intestinal microbiome, playing a vital role in maintaining host health and homeostasis. Microbial residents in the human intestine actively contribute to the production and synthesis of essential compounds, such as vitamins, amino acids, short-chain fatty acids, and various metabolites through intricate enzymatic activities and metabolic pathways. A significant portion of these metabolites form the necessary foundation for the activities of epigenetic enzymes. These biochemical processes are integral to functions like food digestion, xenobiotic metabolism, and the composition of bioactive molecules. Disruption of this delicate balance, termed microbial dysbiosis, is characterized by a diminished diversity of bacterial species within the intestinal microflora and a decline in beneficial bacteria. Such dysbiosis can compromise intestinal permeability, leading to an altered microbial landscape that may contribute to increased permeability. Consequently, this disruption adversely affects nutrient absorption, food metabolization, and immune system modulation. The resulting perturbations in the intestinal microflora have the potential to precipitate complications and influence the development of various pathological conditions [7,8].

This comprehensive review aims to explore the intricate mechanisms influencing the composition of the intestinal microbiota throughout the aging process. Furthermore, it will investigate the potential of polyphenols as a strategic tool to foster the preservation of a robust and balanced intestinal microbiota.

## 2. Microbiota Dynamics in the Aging Process

The intestinal microbiota comprises microorganisms inhabiting the human intestine and undergoes changes with age. From birth, the intestinal microbiota aids in the maturation of the immune system and contributes to homeostasis. Studies in both humans and animal models suggest microbial alterations in the aging intestine, even under healthy conditions [9,10,11]. The population of bacterial phyla, comprising over 90% of the intestinal microbiota, is principally composed of *Bacteroidetes* and *Firmicutes*. The remaining phyla, present in lesser abundance, comprise diverse species, some of which may contribute metabolites and essential functions to facilitate healthy aging [12].

The microbiota composition varies within individuals across different segments of the gastrointestinal tract, particularly between the cecum and the rectum. Additionally, there is a noticeable gradient in diversity and bacterial load from the oropharynx to the jejunum, with a prevalence of *Proteobacteria* and *Firmicutes*. Conversely, an increase in bacterial load is observed from the ileum to the colon, characterized by a predominance of *Firmicutes*, *Bacteroidetes*, and *Proteobacteria*. Reports suggest that the microbiota may exhibit greater interindividual variability in bacterial composition, and variations between segments within an individual can lead to differences in the metabolism of dietary molecules and medications. These variations contribute to an increased susceptibility to various diseases [13,14,15,16,17].

Throughout life, changes in the diversity of the intestinal microbiota occur. Naturally, in early life, the intestinal microbiota is well established, but subtle variations persist until around the age of 40. A certain stability is achieved during this period, but it can be lost over the aging process due to the aging of prokaryotic and eukaryotic symbionts, resulting in a dysbiosis state [18,19,20,21].

After the age of 70, noticeable alterations in the composition of the intestinal microbiota occur as a consequence of the cumulative impact of various stressors, the physiological aging processes within the gastrointestinal system, and shifts in lifestyle and dietary habits. These changes typically manifest as a reduction in microbial biodiversity, accompanied by an increase in opportunistic Gram-negative bacteria and a decrease in species purportedly associated with health-promoting functions [9,22,23].

Alongside changes in microbiota diversity, aging also results in a reduction in the microbiota’s metabolic capacity, lower levels of short-chain fatty acids, and potential connections with age-related conditions. These may include disruptions in intestinal transit, weight loss, reduced appetite, cognitive decline, frailty, vitamin D deficiency, hypertension, diabetes, arthritis, sarcopenia, and more [24,25,26]. Moreover, during the aging process, there is a decline in the function of the intestinal barrier modulated by intestinal microbiota, leading to a decrease in tight junction proteins and mucins production. Research has indicated that the presence of *A. muciniphila* is diminished in individuals with age-associated disorders compared to their healthy counterparts of the same age, underscoring the potential benefits of supplementing with *A. muciniphila* to enhance the thickness of colonic mucus. Age-related intestinal disorders have been observed to reduce the thickness of colonic mucus [27,28]. In addition, a probiotic combination featuring strains of *Lactobacillus* and *Enterococcus* isolated from healthy infants, when administered to elderly mice with dysbiosis, facilitated a reduction in intestinal permeability. This effect was achieved through an increase in bile salt hydrolase activity, inducing a taurine-dependent expression of tight junction proteins (Zo-1 and occludin). These findings suggest that microbiota-based therapies hold promise for potentially reversing the age-related deterioration in intestinal barrier function [29,30,31].

Intestinal commensal bacteria exert a substantial influence on individual nutrition, metabolism, and the production of microbiota-derived metabolites. Notably, short-chain fatty acids are promptly absorbed into the plasma through the individual’s intestinal epithelium. Emerging studies propose that interventions based on microbiota-derived metabolites may positively impact healthy aging, as these metabolites demonstrate the potential to influence human longevity [32,33,34,35,36]. Noteworthy findings indicate that the suppression of folate metabolism entails anti-aging effects in individuals. Intriguingly, this effect appears to be indirectly mediated by bacterial influence, underscoring the pivotal role of the microbiota and microbial metabolites in human health. This underscores the importance of exploring strategies aimed at extending longevity, particularly given that elderly individuals often exhibit elevated serum levels of inflammatory cytokines (IL-6) and c-reactive protein (CRP) [37,38,39,40,41,42].

Human studies reveal a decline in the population of microorganisms within the aging intestinal microbiota, characterized by reduced *Firmicutes* and *Bifidobacteria*. Concurrently, there is an elevation in *Bacteroidetes* and pro-inflammatory *Enterobacteriaceae*. Functionally, the microbiota of elderly individuals diverges from that of their younger counterparts; notably, young individuals exhibit a higher production of short-chain fatty acids compared to the elderly [43,44,45,46], which in turns helps the immune system.

The aging process is intricately connected to changes in the immune system, known as immunosenescence, and inflammation. Immunosenescence involves a decline in immune functions, affecting the ability to combat infections and respond to new antigens. This condition also contributes to increased autoimmunity and inefficient wound healing. Simultaneously, inflammation often results from immunosenescence, marked by reduced serum levels of anti-inflammatory cytokines like IL-10, along with a decrease in pro-inflammatory cytokines such as IL-1β, IL-6, IL-8, and TNF-α. These immunological and inflammatory alterations play crucial roles in shaping the aging immune system and its responses [47].

The aging process induces alterations in the intestinal microbiota, disrupting innate immunity balance. This is linked to an increased proliferation and redistribution of myeloid cells, showcasing a significant elevation in peripheral-origin antigen-presenting cells (APCs) in the brain. The accumulation of myeloid cells associated with aging may positively correlate with changes in the intestinal microbiota, including a reduction in *Akkermansia* and behavioral deficits [48]. As aging advances, the functions of chemotaxis, phagocytosis, and the production of reactive oxygen species (ROS) may suffer impairment. Nonetheless, the proportions of circulating neutrophils do not appear to be affected. Animal models demonstrate improved survival in endotoxin-induced septic shock. The microbiota influences neutrophil aging through Toll-like receptor (TLR) and MyD88 signaling. In animal models using germ-free (GF) mice or those treated with antibiotics, microbiota depletion reduced the number of circulating aged neutrophils, mediated by decreased CXCR4 and increased CD68 [49].

**Figure 1:**
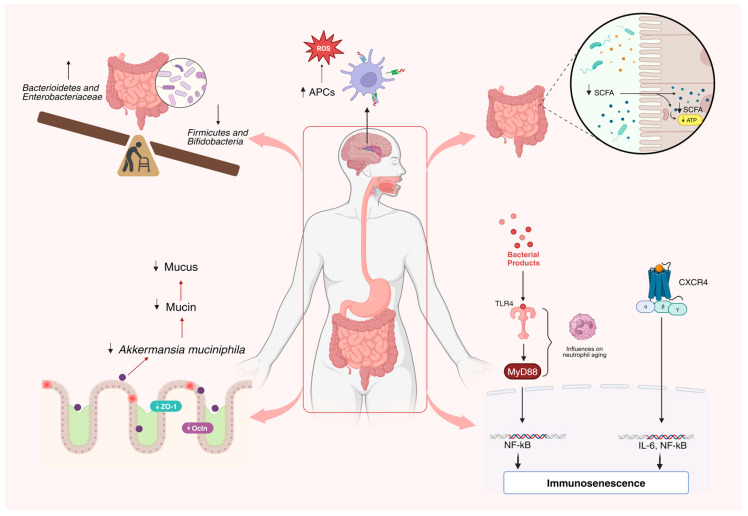
Impact of aging on the composition of the intestinal microbiota. Significant changes in the intestinal microbiota composition arise from various stress factors linked to aging, resulting in a dysbiotic state. This imbalance entails reduced microbial diversity, fostering an uptick in opportunistic Gram-negative bacteria while decreasing species associated with health benefits. Furthermore, these shifts disrupt innate immunity, notably increasing antigen-presenting cells in the brain. Myeloid cell buildup reduces *Akkermansia muciniphila*, diminishing colonic mucus and heightening IL6-mediated inflammation. Additionally, alterations in tight junction expression (Zo-1 and occludin) contribute to changes in intestinal permeability [9,12,22,23,29,30,31,47,48].

It is crucial to emphasize the coordinated regulation of the commensal microbiota, the preservation of the epithelial barrier, and the controlled secretion and re-absorption of enzymes and nutrients. The dysregulation of these homeostatic mechanisms results in an imbalance, promoting intestinal dysbiosis and chronic inflammation. Consequently, this heightens the risk of diseases and negatively impacts epithelial regeneration, potentially leading to the development of dysplasias and cancers [50,51]. Recognizing the intricate interactions involved in intestinal regeneration, encompassing epithelial functions, the commensal microbiota, and the immune system, is crucial. Exploring strategies for intervening and preventing gastrointestinal diseases is imperative, especially considering that aging is a significant risk factor for various age-related conditions, including chronic and inflammatory diseases, not to mention those affecting the gastrointestinal tract. The elderly population becomes more susceptible to inflammatory and infectious diseases [50] (Figure 1).

## 3. Impact of the Gut Microbiota on Age-Associated Diseases

**Figure 2:**
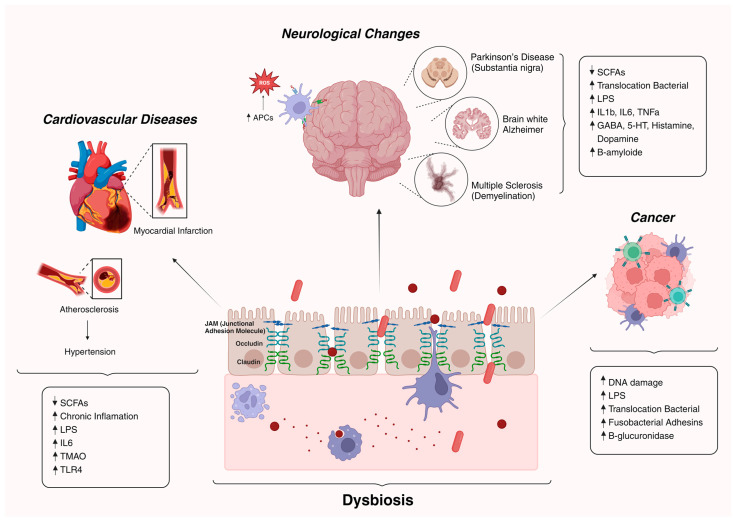
Diseases associated with age promoted by dysbiotic condition. Changes in the intestinal microbiota during the aging process significantly impact health, heightening vulnerability to age-associated ailments such as neurodegenerative and cardiovascular afflictions, alongside conditions conducive to cancer development [4].

The intestinal microbiota significantly influences overall health through complex bidirectional signaling with the central and peripheral nervous systems and other distant organs. Disruption of this intricate communication between bacteria and the host leads to dysbiosis, thereby fostering the development of various diseases, including neurological disorders, cardiovascular conditions, and cancer, among others [4] (Figure 2).

### 3.1. Neurological Changes and Dysbiosis

Communication involving the brain–gut axis is associated with metabolic, endocrine, neural, and immune pathways, which can act independently or cooperatively [34]. Detrimental alterations in the gut microbiota are not intrinsically linked to the aging process. Some evidence suggests that when individuals are healthy, age may not significantly impact the composition of the gut microbiota, except during phases of rapid neurodevelopment (such as late adolescence and emerging adulthood). Across both young and older adulthood, there is considerable variability in the microbiota among individuals, yet there is relative stability within an individual [52]. The role of the intestinal microbiota in modulating central physiological and pathological processes is based on the ability of bacteria to produce bioactive products that provide insight into the mechanisms involved [34].

Increased intestinal permeability may result from a decrease in short-chain fatty acids (SCFAs), inducing cell apoptosis and disrupting the expression of tight junction proteins. This disruption facilitates the translocation of bacterial products, including LPS, microbial toxins, and even whole bacterial cells into circulating tissues. Consequently, there is heightened transport of bacterial metabolites to the brain, leading to increased neuroinflammation and further promoting the dysbiotic state. Beyond the intestinal barrier, dysbiosis is associated with the breakdown of the blood–brain barrier. This can exacerbate the reduction in intestinal integrity, triggering systemic inflammation correlated with various metabolic, neuroinflammatory, neuropsychiatric, and neurodevelopmental or neurodegenerative conditions. Elevated levels of circulating pro-inflammatory cytokines such as IL-1β, IL-6, TNF-α, and CRP (C-reactive protein) characterize these associations [53,54,55,56,57,58]. Furthermore, several studies indicate that peripheral inflammation can compromise the integrity of the blood–brain barrier, enabling the spread of inflammation to the central nervous system. Additionally, there were adverse effects on tight junction proteins like Occludin and Claudin-5, coupled with an upregulation of caspase-3 levels in the cortex and hippocampus. This implies that the apoptotic cascade triggered by the inflammatory process and oxidative stress could contribute to neuronal death in specific brain regions [59].

The link between brain interaction and bacterial products influencing behavior is intriguing. Various genera and species of bacteria can produce metabolites involved in mood-related functions and behavioral and cognitive functions, acting as neurotransmitters or precursors of neurotransmitters such as gamma-aminobutyric acid (GABA), serotonin (5-HT), histamine, and dopamine [60,61]. For instance, *Bifidobacterium infantis* increases tryptophan levels in blood plasma, affecting central serotonin transmission. GABA can be produced by *Lactobacillus* and *Bifidobacterium*; norepinephrine by *Escherichia*, *Bacillus*, and *Saccharomyces* species; serotonin by *Streptococcus*, *Candida*, *Escherichia*, and *Enterococcus* species; dopamine by various bacteria; and acetylcholine by *Lactobacillus* [62].

Metabolic functions involving the individual and intestinal microbiota encompass the production of essential metabolites like short-chain fatty acids. These include acetate, propionate, and butyrate, which operate through G protein-coupled receptors or histone deacetylases. These metabolites play a crucial role in brain–gut communication and may represent potential therapeutic targets for neurodevelopmental and neurodegenerative disorders, as well as in the progression of neurological changes [63,64].

The gradual accumulation of abnormal proteins in the central nervous system may characterize the development of neurological disorders, including Alzheimer’s disease (AD), Parkinson’s disease (PD), and Multiple Sclerosis (MS) [65]. AD is a progressive neurodegenerative syndrome linked to the buildup of misfolded amyloid-β (Aβ) protein fibrils and oligomers. Additionally, there are neurofibrillary tangles composed of hyperphosphorylated tau protein found in the cerebral cortex and other brain regions [66,67]. 

An elevated *Firmicutes*/*Bacteroidetes* ratio characterizes intestinal dysbiosis, potentially leading to the accumulation of amyloid precursor proteins from the early stages of Alzheimer’s disease (AD) [68]. A study found increased specific bacterial populations, including *Actinomycetes* and *Bacteroides*, in the intestines of Alzheimer’s disease (AD) patients compared to the control group [69]. This rise in pathogenic bacteria, along with elevated *E. coli*/*Shigella*, is linked to heightened pro-inflammatory cytokines (interleukin IL-1 and CXCL2) expression and NLRP3 inflammasome activation [70]. Clinical research revealed lower levels of anti-inflammatory *Bacteroides fragilis* and *Eubacterium rectale* in the intestinal tract of elderly individuals with cognitive impairment, while pro-inflammatory bacteria like *E. coli* and *Shigella* were more prevalent [71,72]. Strains such as *Bacillus subtilis*, *E. coli*, *Salmonella*, *Mycobacterium*, *Mycobacterium tuberculosis*, and *Staphylococcus aureus* produced functional extracellular amyloid fibers [66]. Certain microorganisms released LPS, amyloid, and other immunogenic mixtures into the surrounding environment, promoting Aβ accumulation and inflammation, activating the signaling pathway in AD pathogenesis. Bacterial amyloid protein is associated with molecular and cellular adaptation, adhesion stimulation, aggregation, biofilm formation, tissue invasion, bacterial colonization, and pathogen infectivity [73]. Conversely, treatment with *Clostridium butyricum* improved cognitive function in rats, reducing neuronal apoptosis, increasing brain-derived neurotrophic factors, and decreasing pro-inflammatory cytokines [74].

PD is a progressive neurodegenerative condition characterized by the accumulation of α-synuclein (α-syn) in dopaminergic nerve cells within the substantia nigra region of the brain. This process leads to the formation of Lewy bodies, which are round lamellated eosinophilic cytoplasmic inclusions. Despite ongoing research, the precise mechanisms underlying PD pathogenesis remain unclear and are likely multifactorial in nature, giving rise to various theories about its origin [75].

Aging significantly increases the risk of developing and progressing PD, impacting multiple cellular pathways and contributing to neurodegeneration [76]. The molecular perturbations tolerated by young neurons may have more severe consequences in aged neurons [77]. Investigations suggest a correlation between gastrointestinal abnormalities in individuals with PD and intestinal dysbiosis, along with α-synuclein deposits in the Enteric Nervous System (ENS) [78,79,80,81]. Research exploring the gut microbiome in PD observed a significant reduction in *Prevotellaceae* species, rather than relative *Enterobacteriaceae* counts, in fecal samples from individuals with PD [82]. Intestinal dysbiosis associated with PD, leading to increased *Akkermansia* and decreased SCFA-producing bacteria, may cause intestinal permeability and inflammation. This, in turn, exposes the intestinal neural plexus to toxins like lipopolysaccharides (LPS) and pesticides, potentially triggering the abnormal aggregation of α-synuclein fibrils and the formation of Lewy bodies [82]. Patients with PD display a distinct microbial composition compared to healthy individuals or those with other neurological disorders [83,84,85].

The intestinal microbiota in PD patients shows a reduced presence of bacteria producing SCFAs, particularly butyrate, such as *Faecalibacterium prausnitzii* and *Lachnospiraceae*, known for their anti-inflammatory properties [86,87,88]. Additionally, a murine model study suggested that certain bacterial species, like *Proteus mirabilis*, may contribute to the development of motor deficiencies and potentially play a role in PD [89]. The gut microbiota also influences the metabolism of medications used to alleviate PD symptoms. For instance, the primary PD drug, levodopa (L-dopa), undergoes decarboxylation to dopamine by tyrosine decarboxylase from *Enterococcus faecalis* and is converted to metatyramine by dopamine dehydroxylase from *Eggerthella lenta* A2 [90,91]. Moreover, L-dopa accumulates on the surface of *Helicobacter pylori* in vitro, possibly affecting the L-dopa concentration in PD patients and leading to motor symptom fluctuations [92,93].

MS is a chronic immune-mediated neurological disease affecting the central nervous system (CNS), causing damage to axons and demyelination, and impacting around 2.3 million people worldwide. The pathophysiology of MS is intricate and not fully understood [94,95]. Its incidence is higher in women [96,97,98]. The primary pathogenic feature involves the development of demyelinated inflammatory plaques in the CNS, affecting gray or white matter in the spinal cord and brain. This triggers a neuroinflammatory response, leading to the demyelination of specialized cells, including oligodendrocytes, and subsequent neurodegeneration. The abnormal permeability of the blood–brain barrier (BBB) allows immune system cells to infiltrate CNS neuronal cells, initiating demyelination. Myelin antigen-specific T cells (CD8+ and CD4+ T cells) cross the BBB, contributing to events that result in the formation of demyelinating lesions [99,100,101]. The gut microbiota influences and modulates the balance between pro- and anti-inflammatory T cells in gut-associated lymphoid tissue [102,103]. Studies have characterized the gut microbiota profile of patients with various forms of MS [104,105,106].

It has been reported that there is a reduction in bacteria from the Actinobacteria phylum, such as Collinsella [107,108], Slackia [108], and Adlercreutzia [107], in MS patients. Conversely, other groups reported an increase in *Bifidobacterium* [109,110] and *Eggerthella* [109] in MS patients. Regarding the *Bacteroidetes* phylum, research indicated a decrease in species like *Prevotella* [107,108,109,111,112], *Parabacteroides* [107,111], *Butyricimonas* [108], and *Bacteroides* [109] in MS patients. Bacteria from the *Firmicutes* phylum, including *Blautia* and *Dorea* (from the *Lachnospiraceae family*) [107], *Streptococcus* [109,112], and *Ruminococcus* [113], were found in higher abundance in MS patients. Conversely, species like *Lactobacillus*, *Coprobacillus*, *Erysipelotrichaceae*, and *Veillonellaceae* [107], as well as *Lachnospiraceae*, *Ruminococcaceae* [110], and *Clostridia* [109,113], were reduced in these patients. Concerning the *Proteobacteria* phylum, an increase in the abundance of *Pseudomonas*, *Mycoplana*, *Haemophilus* [107], *Bilophila* [110], and *Acinetobacter* [110] was observed in MS patients, while the presence of *Sutterella* was decreased [109]. Interestingly, one study reported an increase in *Sutterella* in MS patients undergoing disease-modifying therapies [108]. Additionally, studies have noted an increase in the abundance of *Akkermansia* species, belonging to the *Verrucomicrobia* phylum, in MS patients [108,111,114].

Based on these findings, it can be posited that the intestinal microbiota holds promise for research in the realm of neurological disorders, particularly neurodegenerative diseases. Moreover, the intricate interactions between gut microorganisms and medications currently employed in the treatment of neurodegenerative conditions require further investigation. A more in-depth exploration of the pathological mechanisms associated with dysbiosis can significantly enhance advancements in early diagnosis, amplify the therapeutic effects of existing medications, and foster the development of new therapeutic targets and drugs for neurodegeneration treatment.

### 3.2. Cardiovascular Diseases and Dysbiosis

Cardiovascular diseases (CVDs) stand out as the primary cause of global mortality, including conditions such as coronary artery disease, cerebrovascular disease, and peripheral arterial disease. The established risk factors for CVDs encompass atherosclerosis, hypertension, diabetes, dyslipidemia, obesity, and mental health disorders. Emerging research suggests a crucial role for the intestinal microbiota in maintaining cardiovascular health, with dysbiosis potentially contributing to the initiation and progression of cardiovascular diseases [115]. For instance, investigations reveal a clear positive correlation of *Atopobium* with various anthropometric variables, such as waist circumference, weight, and body mass index, as well as with fat and protein intake reported in a 24-h dietary recall study [116]. Moreover, metagenomic studies have demonstrated a positive correlation between Clostridium and the formation of metabolites like trimethylamine N-oxide (TMAO), along with a positive correlation between *Clostridium histolyticum* and *Clostridium perfringens* and waist circumference, weight, body mass index, and fat mass. This suggests that both the aforementioned *Clostridium* and *Atopobium* species can be considered markers of inflammation and CVD risk [116,117].

The intestinal microbiota actively participates in the metabolism of choline, phosphatidylcholine, and carnitine, key compounds implicated in the production of metabolites such as TMAO. Scientific evidence indicates that TMAO not only influences cholesterol and bile acid regulation but also correlates with early-stage atherosclerosis and an increased long-term risk of cardiovascular disease-related mortality [118]. Additionally, studies indicate that metabolites from the intestinal microbiota can contribute not only to inflammation but also to the development of high blood pressure [119]. Furthermore, the overproduction of metabolites such as ammonia and ammonium hydroxide, derived from the fermentation of proteins by the intestinal microbiota, disrupts the tight junctions between intestinal epithelial cells. This results in further increases in microbial translocation and systemic inflammation [120,121].

Some studies have explored the link between gut microbiota and markers of low-grade chronic inflammation in humans [122,123,124,125,126,127]. A systematic review of human studies revealed that the abundances of *Faecalibacterium*, *Bifidobacterium*, *Ruminococcus*, and *Prevotella* are inversely associated with various markers of low-grade inflammation, including high-sensitivity C-reactive protein and interleukin (IL)-6. Understanding the connections between the intestinal microbiota and markers of low-grade inflammation in humans holds promise for developing therapeutic strategies to prevent and treat atherosclerotic cardiovascular disease, considering the intestinal microbiota’s interaction with the innate and adaptive immune systems [57]. Similarly, another investigation found a contrasting association between *Prevotella* and inflammatory markers. An increased abundance of specific *Prevotella* species was linked to low-grade inflammation in systemic diseases like rheumatoid arthritis [127]. The study also noted that individuals with obesity exhibited a lower abundance of *Prevotella* species in the intestine. Furthermore, *Prevotella* abundance showed an inverse association with lipopolysaccharide (LPS) and high-sensitivity C-reactive protein [127].

LPS, a known inflammatory mediator found in Gram-negative bacteria, is primarily distributed in the intestine and oral cavity. Studies have demonstrated that LPS can induce vascular oxidative stress by activating Toll-like receptor 4 (TLR4), resulting in endothelial dysfunction and vascular inflammation. Notably, individuals with cardiovascular diseases exhibit elevated fecal levels of LPS compared to their healthy counterparts. It is worth mentioning that the activity of LPS can be discerned based on the distinct lipid A structures of LPS originating from different bacterial sources [128].

An increased presence of opportunistic pathogens originating from the host, such as *Escherichia coli*, *Clostridium ramosum*, *Bacteroides caccae*, and *Eggerthella lenta*, along with a decrease in bacteria that synthesize short-chain fatty acids (SCFAs) like *Roseburia*, *Faecalibacterium*, and *Eubacterium rectale*, has been associated with a higher risk of developing cardiovascular diseases. This highlights that both the types and abundance of microorganisms in the intestinal microbiota serve as risk factors influencing the development of cardiovascular diseases [129,130].

In patients at high risk of stroke, there is a reduction in butyrate-producing bacteria, such as those from the *Lachnospiraceae* and *Ruminococcaceae* families. This reduction leads to decreased fecal butyrate levels and simultaneous increases in intestinal pathogens, particularly those from the *Enterobacteriaceae* and *Veillonellaceae* families [131]. Moreover, there is an observed rise in the prevalence of opportunistic pathogens like *Megasphaera*, *Enterobacter*, and *Clostridium difficile* [132,133,134]. In a post-stroke clinical study, a decrease in the abundance of *Roseburia*, *Bacteroides*, and *Faecalibacterium prausnitzii* was noted, accompanied by an increase in *Enterobacteriaceae*, *Bifidobacteriaceae*, and *Clostridium difficile* [135].

Finally, atheroma plaques from patients with coronary artery disease (CAD) have been found to contain pathogenic species such as *Staphylococcus*, *Proteus vulgaris*, *Klebsiella pneumoniae*, and *Streptococcus species* [131]. In these patients, there is an increase in the abundance of *Lactobacillus*, *Streptococcus*, *Escherichia*, *Shigella*, and *Enterococcus species*, accompanied by a reduction in *Faecalibacterium*, *Subdoligranulum*, *Roseburia*, *Eubacterium rectale*, and *Bacteroides fragilis* species. The latter group is known to regulate T cell functions in the intestinal mucosa, providing anti-inflammatory effects and protecting the intestinal barrier [131,136].

In general, extensive research has been conducted on the involvement of the intestinal microbiota in the development of cardiovascular diseases (CVDs). Understanding these interactions is crucial for enhancing the effectiveness of current CVD therapies, ensuring better suitability and response to treatment, and fostering the development of new prophylactic approaches aimed at cardiovascular health through the manipulation of the intestinal microbiota.

### 3.3. Cancer and Dysbiosis

The intestinal microbiota significantly influences the development and progression of various cancers by modulating metabolic and genetic processes. Microorganisms residing in tissues can increase cancer risk, shape immune responses, and enhance the effectiveness of anticancer treatments [137,138,139,140,141,142,143,144].

The mechanisms through which tissue-resident microbiota may promote cancer development are not fully explored. However, they are associated with inducing inflammatory responses that create a favorable environment for tumors and breaking down tissue barriers, facilitating the translocation of metabolites from the intestinal microbiota. Due to systemic effects, intestinal microbes are implicated in inducing cancer in various organs, including the skin and brain. The dynamic interaction between the resident microbiota and the host involves specific tissue factors, pH alterations, antimicrobial presence, mucin, and bile acids, among others. The microbiota’s impact is mediated by microbial diversity, nutrient availability, competition between species, and adaptive mechanisms [145,146].

It has been reported that gut microbiota’s metabolism can influence cancer progression. The microbiota may exert carcinogenic effects directly or indirectly through its products, involving three key mechanisms in carcinogenesis. Firstly, it can induce DNA damage, alter gene expression, and stimulate proliferation. Secondly, it may promote protumorigenic microbial niches, including biofilm formation. Lastly, imbalances in the immune response can contribute to its carcinogenic impact [147]. Certain bacteria, such as *Escherichia coli*, *Bacteroides fragilis* enterotoxigenica, and *Fusobacterium nucleatum*, have demonstrated virulence factors linked to colorectal cancer development. Fusobacterial adhesins like Fap2, RadD, and FadA, expressed by *Fusobacterium nucleatum*, contribute to bacterial aggregation, adhesion to dysplastic tissues, and biofilm formation [148]. Colicin B2, produced by *E. coli*, induces DNA damage [149,150], while CagA, a cytotoxin from *Helicobacter pylori*, known for inducing inflammatory pathways in gastric cancer, may also play a role in colorectal cancer tumorigenesis [151]. Additionally, recent research has unveiled insights into the metabolites of the intestinal microbiota. Studies indicate that gut microbial β-glucuronidase (βG) found in specific strains is implicated in promoting azoxymethane-induced gut microbial dysbiosis and intestinal tumorigenesis [152,153].

The intestinal microbiota can impact tumor development beyond the gastrointestinal tract [154]. Evidence indicates that intestinal bacteria can travel to adjacent tissues, influencing tumor progression. For instance, a study using fluorescence-labeled commensal bacteria in a murine model demonstrated bacterial translocation from the intestine’s luminal compartment to the pancreas [155]. It is proposed that bacteria, like *Gammaproteobacteria*, translocate from the intestine to pancreatic tumors, metabolizing the active form of the drug gemcitabine and diminishing its effectiveness. The suggested route for this translocation is likely through the pancreatic duct, which communicates with the duodenum [156].

Moreover, it is theorized that molecules such as metabolites, antigens from intestinal bacteria, and pathogen-associated molecular patterns can be transported to the liver through the hepatic portal vein. Given that about 70% of the liver’s blood supply comes from the intestinal circulation, the liver becomes a crucial site for interactions between signals from the intestinal microbiota and the immune system. Studies indicate that molecules from intestinal bacteria, including secondary bile acids (BAs), lipopolysaccharide (LPS), and lipoteichoic acid (LTA), can either drive carcinogenesis or suppress antitumor immunity in the liver [30,157,158,159].

These findings highlight the profound impact of the intestinal microbiota on the human immune system and its recognition as a crucial factor in modulating antitumor immunity and therapeutic outcomes. Monitoring the microbiological composition of this organ could aid in identifying individuals at a higher risk of developing tumors, and modulating the microbiota in these individuals may have implications for cancer prevention. Recent studies revealing the presence of intestinal bacterial species in previously considered sterile tissues underscore the need for the further exploration of microbial colonization sources and the composition and function of the microbiota in these locations.

## 4. Polyphenolic Compounds and Their Significance in Shaping the Intestinal Microbiota

The preservation of a healthy intestinal microbiota, where the predominance of beneficial bacteria surpasses that of pathogenic ones, engenders various health advantages and supports immune functions. Nevertheless, several endogenous and exogenous factors, including nutritional status, age, genetic conditions, and immune status, can perturb the intestinal equilibrium, leading to the onset of gastrointestinal disorders such as irritable bowel syndrome, antibiotic-associated diarrhea, and inflammatory bowel disease [160,161]. Much research evidence suggests that polyphenols, secondary metabolites derived from the metabolism of vegetables and fruits, possess health-promoting properties and contribute to the prevention of chronic diseases such as coronary heart disease, type 2 diabetes, neurodegenerative diseases, and certain types of cancer [162,163]. Evidence indicates that the prolonged daily intake of polyphenols can modulate the intestinal microbiota, fostering a beneficial microbial ecosystem that significantly enhances human health. It has been established that polyphenol consumption induces alterations in various aspects of the intestinal microbiota composition, including changes in the abundance of Bifidobacterium, Proteobacteria, and Helicobacter [164]. Moreover, experimental studies conducted in both pre-clinical and clinical phases suggest that polyphenolic compounds play a pivotal role in augmenting beneficial intestinal bacteria, such as *Akkermansia muciniphila*, while concurrently reducing the prevalence of pathogenic microbial species such as *Flexispira*, *Prevotella*, *Adlercreutzia*, *Dessulfovibrio*, *Bifidobacterium*, *Lactobacillus*, *Deferribacteres*, and *Actinobacteria* [165,166].

Polyphenols exert their antimicrobial effects through diverse mechanisms, which can vary depending on the structural differences in the cell membranes of Gram-positive and Gram-negative bacteria. The concentration-dependent binding of polyphenols to bacterial cell membranes alters membrane functionality, impeding bacterial growth. This interaction, particularly with catechins, induces the production of H_2_O_2_, affecting the permeability of microbial cell membranes. Notably, this phenomenon is observed across various bacterial species, including *Bordetella bronchiseptica*, *E. coli*, *Klebsiella pneumoniae*, *Serratia marcescens*, *Pseudomonas aeruginosa*, *Salmonella choleraesuis*, *Bacillus subtilis*, and *Staphylococcus aureus*. Moreover, polyphenols, such as epicatechin gallate, have demonstrated the ability to sensitize bacteria to the effects of antibiotics, a promising aspect for addressing antibiotic-resistant strains, as evidenced in methicillin-resistant S. aureus treated with beta-lactam antibiotics [167].

In vitro investigations have underscored curcumin’s potential as a compound for rectifying disrupted intestinal permeability. Employing CaCo_2_ cell studies, curcumin showcased inhibitory effects on the disruption of the intestinal epithelial barrier, neutralized IL-1β secretion induced by LPS, and prevented occludin junction protein disruption [168,169]. Noteworthy actions of curcumin included the inhibition of IL-1β-induced MAPK p38 activation, the augmentation of phosphorylation in occludin junction proteins, and their preservation of their normal arrangement [169]. In vivo assessments in rat models further affirmed curcumin’s efficacy, improving tight junction structure, suppressing serum TNF-α and LPS concentrations, and positively modulating occludin expression in the intestinal mucosa [170]. Another study corroborated these findings, demonstrating that curcumin significantly enhanced intestinal barrier function by restoring alkaline phosphatase activity and the expression of tight junction proteins ZO-1 and claudin-1 [171].

Ohno et al., in an in vivo study utilizing animal models, demonstrated the remarkable anti-inflammatory properties of a newly developed nanoparticle curcumin, a polyphenol derivative, in mice with DSS-induced colitis. The intervention led to a significant reduction in pro-inflammatory mediators, restrained Treg expansion, and elevated fecal butyrate levels. Notably, curcumin effectively suppressed NF-κB activation and modulated the expression of pro-inflammatory mediators within the colonic epithelial cells of treated mice [172]. Additionally, alternative anti-inflammatory mechanisms of curcumin were proposed, including the attenuation of LPS-induced inflammation and inhibition of the TLR4/MyD88/NF-κB signaling pathways [173,174]. The compound exhibited a noteworthy capability to inhibit NF-κB nuclear translocation and downregulate pro-inflammatory genes implicated in cancer pathogenesis [175].

In an in vitro model, flavan-3-ol monomers such as (+)catechin and (-)epicatechin have demonstrated the capacity to enhance the bacterial population in the colon, particularly influencing the growth of *Clostridium coccoides*, *Eubacterium rectale*, and *E. coli* compared to *Lactobacillus* spp. and *Bifidobacterium* spp., which remained unaltered [176]. Moreover, in an animal model of colon cancer, the extract from red wine, rich in proanthocyanidins, was shown to modulate the predominance of bacterial species, favoring *Bacteroides*, *Bifidobacterium*, and *Lactobacillus* spp. over *Bacteroidetes*, *Propionibacterium*, and *Clostridium* spp. [177]. In a separate study utilizing a rodent model of dextran sulfate sodium (DSS)-induced colitis, grape resveratrol was found to stimulate the fecal cell count of *Lactobacillus* and *Bifidobacterium* spp. [178].

A study conducted on healthy adults treated with a grape seed extract rich in proanthocyanidins for two weeks revealed a significant increase in *Bifidobacteria* [179]. In vivo investigations have suggested that monomeric flavan-3-ols and abundant sources of flavan-3-ols, such as grape seed extract, green tea, currants, and chocolate, possess the ability to modulate the intestinal microbiota, inducing alterations in beneficial bacteria like *Lactobacillus* spp. However, both in vitro and in vivo studies have demonstrated the inhibition of other groups, such as *Clostridium* spp. [176,180,181,182].

## 5. Polyphenolic Compounds, Metabolization, and Their Significance in Modulating Intestinal Microbiota during Aging

The metabolization of polyphenols, a critical process for unlocking their bioactive potential, begins as these compounds enter the intricate pathways of the human digestive system. The efficiency of polyphenol absorption is influenced by various physicochemical factors, including the size of the molecule, the presence of functional groups, its spherical shape, lipophilicity, and solubility [183]. Once absorbed, the phenolic compounds are the subject of intense metabolization, which leads to metabolites with more bioavailability than the phenolic compounds [184].

Polyphenols, once ingested, are processed through a series of metabolic reactions within the gastrointestinal system [185,186]. These compounds are initially metabolized in the intestine (predominantly phase II reactions) and the liver (both phase I and II reactions) [187,188]. The liver’s phase I metabolism is primarily facilitated by the cytochrome P450 enzymes. In the intestine, enzymes like lactase-phlorizin hydrolase and cytosolic β-glucosidase transform these compounds into forms that can be readily absorbed [186]. These forms are further modified through glucuronidation, sulfation, and methylation, depending on the species inhabiting the intestine, enhancing their solubility and thus enabling their elimination through bile and urine [188]. Furthermore, more than 90% of dietary polyphenols remain unabsorbed in the small intestine, along with hydrophilic conjugates formed in the liver, which are transported to the colon, where they encounter diverse bacteria capable of extensive metabolism [186,189]. These bacteria have enzymes capable of breaking down various polyphenol structures, including the separation of sugar molecules from aglycones and the breakdown of complex rings within the polyphenol structures. The gut microflora also processes conjugates by detaching sugars and sulfates, converting them back into their aglycone form [188]. These microbes play a crucial role in converting polyphenols into low-molecular-weight phenolic metabolites, which exhibit improved bioavailability and health effects. By acting in a “prebiotic-like” manner, polyphenols can modify the composition of gut microbiota, favoring the growth of beneficial strains like *Bifidobacterium* and *Lactobacilli* while reducing the population of pathogens such as *Clostridium perfringens* and *Clostridium histolyticum* [190]. Notably, the metabolism of polyphenols by gut microbiota varies among individuals due to differences in microbiota composition, leading to diverse health outcomes even with similar daily polyphenol intake [191]. It is equally important that these mechanisms are crucial as they limit any potential toxic effects of polyphenols and also regulate their bioavailability within the body [192].

The onset and advancement of age-related diseases are closely linked to lifestyle and diet. There is a rising interest in polyphenol-rich foods due to their potential to safeguard neurons from damage, alleviate neuroinflammation, and enhance cognitive functions, learning, and memory [193].

An aging model study based on in silico prediction and in vitro transport studies across endothelial cells of the blood–brain barrier, at circulating concentrations, evaluating the potential of known bioavailable phenolic sulfates, arising from the colonic metabolism of berries, provided evidence of differential transport, probably related to the chemical structure, observed that the metabolites of polyphenolic compounds can exert neuroprotective effects when reaching the brain, crossing the blood–brain barrier, observing that preconditioning with phenolic sulfates favored an improvement in cellular responses to oxidative, excitotoxic, and inflammatory injuries, attributing that this attenuation of neuroinflammation was achieved through the modulation of the NF-κB pathway [194].

Bondonno and colleagues in a randomized clinical trial model with healthy individuals observed that the use of apples rich in flavonoids plays a role in the vasodilatory response, attributed to the abundance of quercetin and (-)-epicatechin in apples, improving endothelial function, reducing blood pressure, and providing benefits to cardiovascular health [195]. Some data related to the benefits of polyphenols in modulating the microbiota are still limited; however, these data allow us to raise hypotheses that these abundant flavonoids in apples may offer benefits to cardiovascular health in elderly individuals, since this age group represents an increased risk for changes in the circulatory system [196].

Saccon et al. conducted a comprehensive investigation into the impact of a senolytic drug combination, namely Dasatinib and Quercetin (D+Q), recognized for its efficacy in diminishing senescent cell burden in aged mice. Employing an aged Balb/c female mouse model, the study aimed to discern the treatment’s effects on senescence markers, inflammatory processes, and the microbial milieu within the intestinal tract. The findings unveiled significant alterations in the expression of senescence-associated genes, such as p16 and p21, alongside inflammatory genes, including *IL-1β*, *IL-6*, *TNF-α*, *Mcp1*, and *Cxcl1*, discerned in both the large and small intestines. Moreover, a discernable shift in the intestinal microbiota was noted, characterized by an augmentation of *Firmicutes* coupled with a diminution of *Verrucomicrobia* across all samples. Intriguingly, an elevated Gram-positive to Gram-negative taxa ratio was identified specifically in the ileum but not in the descending segments of the intestine, namely the cecum and colon. The nuanced microbiome signatures observed in conjunction with the amelioration of senescence and inflammation post-senolytic treatment highlight intricate associations in the intestinal ecosystems of aged mice [197].

It is known that curcumin possesses anti-inflammatory, antitumoral, and other beneficial properties [198,199,200,201]. In the context of aging, studies in aged rat models have revealed that curcumin can directly modulate the intestinal microbiota, primarily due to its low bioavailability and absorption. The consumption of curcumin has been demonstrated to induce changes in the bacterial proportions of the intestinal microbiota, favoring an increase in the abundance of *Bifidobacteria* and *Lactobacillus* while reducing the levels of *Prevotellaceae*, *Choriobacteria*, *Enterobacteriaceae*, and *Enterococcus* [202,203,204].

In a study scrutinizing aging-induced alterations in mice, the salutary effects of chlorogenic acid (CGA) and epicatechin-3-gallate (EGCG), principal polyphenolic constituents of coffee and green tea, respectively, were evaluated. Noteworthily, both compounds exhibited discernible intestinal fortification, either in concert or in isolation. The amalgamated administration of CGA+EGCG, harnessing its prowess in antioxidant and anti-inflammatory spheres, coupled with its microbiota-modulating capacities, efficaciously ameliorated cognitive deficits and fortified the intestinal barrier’s functional integrity in comparison to their solitary counterparts. This holistic approach not only thwarted the accrual of D-galactose-induced reactive oxygen species but also augmented the overall oxidative prowess. The supplementation with CGA+EGCG effectively curbed the expression of pro-inflammatory cytokines—namely, TNFα, IFNγ, IL1β, and IL6—culminating in the mitigation of intestinal inflammation. Furthermore, it orchestrated a harmonious modulation of the D-galactose-disrupted intestinal microbiome, orchestrating a restitution of *Firmicutes*/*Bacteroidetes* equilibrium in the aged murine cohort. This intervention intricately orchestrated a reduction in the abundance of *Lactobacilliaceae*, *Erysipelotrichaceae*, and *Derribacteriacea*, concomitant with an elevation in the proportions of *Lachnospiraceae*, *Muribaculaceae*, and *Rikenellaceae* [205].

Diets enriched with polyphenolic compounds have exhibited pronounced efficacy in modulating intestinal permeability and the composition of the gut microbiota in the elderly population. In a clinical study involving elderly subjects subjected to a polyphenol-rich dietary regimen featuring theobromine and methylxanthine derivatives from cocoa and/or green tea, a significant positive correlation was observed between polyphenol-rich diets and the abundance of butyrate-producing bacteria, notably from the *Clostridiales* order and genera *Roseburia*, *Butyricicoccus*, and *Faecalibacterium*. Conversely, an inverse relationship with zonulin levels was noted. Furthermore, a comprehensive multi-omics analysis unveiled direct correlations between specific polyphenol metabolites, namely hydroxyphenylpropionic acid sulfate, 2-methylpyrogallol sulfate, and catechol sulfate, and the abundance of *Butyricicoccus*. Intriguingly, hydroxyphenylpropionic acid sulfate and 2-methylpyrogallol sulfate exhibited a negative correlation with *Methanobrevibacter*. This intricate interplay was underscored by participant age, baseline zonulin concentrations, and shifts in *Porphyromonadaceae* abundance, emerging as pivotal determinants steering the impact of polyphenol-rich diets on zonulin dynamics. These findings postulate that understanding the intricate relationships among polyphenol consumption, intestinal permeability, and the intricate landscape of the gut microbiota in the elderly holds promise for tailoring precision dietary interventions in this demographic [206].

**Figure 3:**
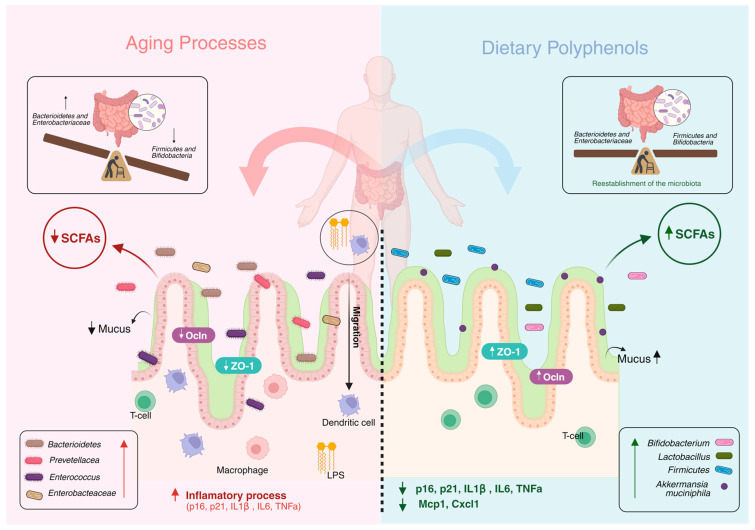
Benefits of polyphenolic compounds in modulating intestinal microbiota. Dysbiosis associated with aging promotes changes in the intestinal microbiota, leading to changes in the proportions of the *Firmicutes*/*Bacterioidetes* phyla, observed by the increase in the proportions of *Pretovellaceae*, *Enterobacteaceae*, and *Enterococcus*, and changes in the intestinal barrier mediated by the reduction in colonic mucus, favoring the development of the inflammatory process. However, the use of polyphenolic compounds confers health benefits mediated by the modulation of the intestinal microbiota, favoring an increase in the proportions of *Fircumutes*/*Bacterioidetes*, *Bifidobacterium*, and *Lactobacillus*, in addition to preventing inflammatory conditions, observed by the inhibition of inflammatory mediator genes such as *p16*, *p21*, *IL1β*, *IL6*, *TNFα*, *Mcp1*, and *Cxcl1*. Polyphenolic compounds favor the increase in the production of SCFA, such as butyrate, and the increase in colonic mucus, which plays an important role in protecting the intestinal barrier [194,195,197,202,203,204,205,206].

The mentioned studies suggest that polyphenolic compounds confer health benefits mediated through the modulation of the gut microbiota, preventing and/or ameliorating inflammatory conditions (Figure 3). Additionally, they are associated with preventing neuroinflammation, inflammatory diseases, cardiovascular diseases, and other age-related ailments (Table 1).

## 6. The Role of Microbiota and Epigenetics during Aging

Recent studies have highlighted that epigenetic changes play a crucial role in how diet and the makeup of gut bacteria impact the body, both in normal and abnormal conditions [214,215,216,217,218,219,220]. This concept, known as the ‘microbiota–nutrient metabolism–host epigenetics axis’, explores how these factors intersect. Epigenetics involves changes passed down through cell division, influencing gene activity through DNA methylation and histone modifications [221,222]. A notable aspect is ‘epigenetic drift’, a phenomenon where DNA methylation patterns change with age. Generally, there is a decrease in methylation in areas with heterochromatin repeats and an increase, or hypermethylation, in promoter CpG regions [223,224]. Histone modifications, including acetylation and methylation, are crucial for cellular aging [225]. Histone methylation levels, regulated by methyltransferases and demethylases, can activate or repress transcription [226]. Gene expression is also influenced by noncovalent epigenetic mechanisms like miRNAs and lncRNAs [227]. These collectively control access to DNA or RNA transcripts, impacting the cell’s transcriptional activities. Epigenetic regulation, shaped by the microbiota, plays a key role in host physiology. This influence occurs through microbial synthesis, regulation of enzymes, and activation of cellular processes affecting epigenetic pathways [218,219,220].

Enzymes like DNA/histone methyltransferases and acetyltransferases modify epigenetics, relying on specific substrates. While the body produces many of these substrates, the microbiota is increasingly recognized as a source. The microbiota generates various compounds influencing epigenetic enzymes [220,228]. Notably, gut microbes produce folate and B vitamins, crucial for DNA or histone methylation. Beneficial microbes like Bifidobacterium and Lactobacillus synthesize folate through one-carbon metabolism, producing S-adenosylmethionine (SAM), a key substrate for DNA and histone methylation [229]. Changes in bacterial composition impact SAM availability, influencing host DNA or histone methylation. Short-chain fatty acids (SCFAs), exclusively produced by gut microbes, result from the fermentation of indigestible carbohydrates. SCFAs inhibit histone deacetylases (HDACs), modifying chromatin and enhancing specific gene expression.

In germ-free mice, both gut and systemic levels of short-chain fatty acids (SCFAs) are notably low [230,231,232]. However, supplementing these mice with SCFAs replicates many transcriptional and epigenetic effects induced by the microbiota [233]. SCFAs not only inhibit HDACs but also influence the tricarboxylic acid (TCA) cycle, impacting enzymes like ten-eleven translocation (TET) methylcytosine dioxygenases, involved in DNA methylation [217]. The microbiota significantly influences DNA methylation patterns in various mammalian cells and tissues [234]. Comparing intestinal epithelial cells (IECs) with germ-free (GF) and conventional (CNV) mice reveals that microbiota exposure leads to DNA hypomethylation and the increased expression of anti-bacterial and anti-inflammatory genes [235]. This decrease in methylation is associated with the heightened activity of DNA demethylase enzymes like Tet3 and Dnmt1 in CNV IECs [236,237]. Deleting Tet3 in IECs increases overall DNA methylation, including in areas sensitive to microbiota changes. In colitis cases induced by dextran sulfate sodium (DSS), CNV IECs show more pronounced hypomethylation compared to GF mice [235].

Human studies have identified correlations between DNA methylation in colon biopsies, microbiota composition, inflammation levels, and diseases like ulcerative colitis (UC) and Crohn’s disease [238]. In UC, linked to colorectal cancer, disease progression correlates with microbial imbalance [239,240]. Specifically, elevated Fusobacterium in UC patients is associated with increased DNA methylation in colorectal-cancer-related genes [241]. Colorectal-cancer-associated microbiota correlates with DNA methylation patterns and induces hyperplasia and DNA methylation in GF mice [240], highlighting the intricate interplay between microbiota and DNA methylation [242].

Microbiota also influence host chromatin through post-transcriptional histone modifications. These modifications, covalently attached to histone tails, crucially alter chromatin structure and regulate gene expression [243]. Gut microbiota impacts the global acetylation and methylation of histones H3 and H4, affecting various tissues (colon, liver, adipose tissue) with diet-dependent variations [243]. Introducing short-chain fatty acids (SCFAs) to germ-free mice partly reinstates histone modifications and gene expression changes influenced by the microbiota [217]. In the intestinal lining, variations in H3K4me3, a histone methylation type linked to active gene transcription, were observed in GF mice compared to conventional mice, resembling patterns found in inflammatory bowel disease (IBD) patients [244]. Gallic acid from the microbiota regulates this modification at Wnt-responsive gene promoters in a colorectal cancer mouse model [245].

Histone deacetylase (HDAC) enzymes, particularly HDAC3, highly expressed in the intestinal epithelium, play a critical role in maintaining intestinal balance dependent on the microbiota [246]. Deleting HDAC3 in intestinal epithelial cells (HDAC3^ΔIEC^) increases H3K9 acetylation, gene expression, impacts Paneth cell balance, and exacerbates dextran sulfate sodium (DSS)-induced inflammation, observed in conventional mice but not in germ-free mice. This highlights HDAC3′s crucial role in normal host–commensal microbe interactions [247].

Research has unveiled the intricate relationship between the host’s epigenome and gut microbiota, a dynamic interplay influenced by dietary choices. The introduction of specific nutrients prompts temporary modifications in the gut microbiome’s composition, thereby intricately impacting the host’s health [248]. Various dietary components, such as fiber, glucosinolates, fats, and polyphenols—processed by gut bacteria—exert profound effects on the host’s epigenetic landscape, shaping outcomes in health and disease [162].

Polyphenols have emerged as focal points for their potential antimicrobial properties and prebiotic attributes, fostering microbial enzyme activities [249]. These activities give rise to compounds capable of eliciting meaningful epigenetic changes in the host [250]. Notably, compounds like curcumin, epigallocatechin gallate (EGCG), and resveratrol derived from plants yield the power to modify the gut microbiota, thereby influencing the host’s epigenetic profile [251]. Curcumin, for instance, stands out for enhancing colon cell function by inhibiting histone deacetylases (HDACs) [252,253]. In a groundbreaking study utilizing an AOM/IL10 knockout model, curcumin demonstrated the ability to prevent the development of colonic tumors, coinciding with an observed increase in microbial diversity [254]. Furthermore, EGCG counteracts metabolic challenges induced by high-fat diets, upregulating DNA methyltransferase 1 (DNMT1) and leading to the reduced methylation of CpG sites in the colon. It also suppresses HDAC activity and diminishes CpG site hypermethylation in colon cancer cells [255]. Resveratrol, through supplementation, induces significant shifts in the composition of gut microbiota in individuals with metabolic syndrome [256]. Additionally, resveratrol-treated mice exhibit heightened Sirtuin 1 expression and a concomitant decrease in pro-inflammatory cytokines, hinting at anti-inflammatory effects [256].

In summary, the intricate interplay between polyphenolic compounds and the gut microbiota stands as a promising advancement, addressing dysbiosis and its associated epigenetic nuances. This area holds potential for pioneering nutritional interventions aimed at combatting the intricate biological effects of aging.

## 7. Conclusions

The aging process triggers biological shifts that influence the dynamics of the intestinal microbiota, potentially reshaping the proportions of essential microbial phyla like Bacteroidetes, Firmicutes, Proteobacteria, and Actinobacteria. The intestinal microbiota serves as a linchpin, orchestrating various physiological and metabolic functions dependent on the delicate equilibrium of bacteria. Factors including lifestyle choices, diet, genetics, epigenetics, and environmental influences contribute to shifts in the intestinal microbiota, laying the groundwork for age-related maladies. Epigenetic modifications, driven by diverse dietary regimes, play a pivotal role in the intricate communication between the gut microbiota and the host’s cellular framework. This emphasizes the growing need to explore effective avenues for modulating the intestinal microbiota to promote health. Enter polyphenol-rich compounds, emerging as stalwart allies in treating and preventing various diseases. Recognized for their anti-inflammatory, antioxidant, and immunomodulatory virtues, polyphenols are increasingly acknowledged for their potential interweaving with the complex dance of the intestinal microbiota in the trajectory of diverse diseases. Moreover, there is a growing focus on how the microbiota can initiate epigenetic adjustments, with polyphenolic compounds potentially holding the key to counteracting these age-related epigenetic changes. While the data in this domain are still evolving, these insights underscore the urgency of delving deeper into the effects of polyphenolic compounds in concert with intestinal microbiota modulation throughout the aging process. This expedition seeks to leverage the functional attributes of bioactive compounds to adeptly modulate and realign processes linked to aging.

## Figures and Tables

**Table 1:** Effects of polyphenols on intestinal microbiota during aging.

Compound	Study Design	*N*	Age	Dose	Intervention Time	Effect	Reference
Grape extracts	In vitro gastrointestinal digestion with *Lactobacillus* and *Bifidobacterium strains*			2 mg/mL	48 h	Prebiotic effect ↑ *Bifodobacteria*	[207]
Resveratrol + H_2_O_2_	Human intestinal cell line Caco-2			H_2_O_2_ (500 μM) plus Resveratrol (80 μM)	24 h	↓ ROS Improved cell viability	[208]
Quercetin + Dasatinib	Female Balb/c aged mice	20	18 months	Dasatinib (5 mg/kg) plus Quercetin (50 mg/kg)	3 consecutive days every 2 weeks over a 10-week period administered by oral gavage	↓ p16, p21, IL1β, IL6, TNFα, Mcp1, Cxcl1 ↑ *Firmicutes* ↓ *Verrucomicrobia*	[190]
Tea polyphenols	Female Sprague Dawley	60	3–4 months	300 mg/kg	12 weeks	Improved intestinal permeability Improved intestinal dysbiosis ↓ TLR4, IRAK, p-IκBα, NF-κB, p65 in the hippocampus ↑ *Bacteroidetes/Firmicutes ratio* ↑ *Bacteroides*, *Lactobacillus*, and *Bifidobacterium*	[209]
Herbal saponins	Male C57BL/6 mice	50	8 weeks old	500 mg/kg	15 days		[210]
Resveratrol	Male Fischer F344 rats	12	Adults	1 mg/kg/day	20 days	↑ *Bifidobacterium* ↑ *Lactobacillus* ↓ Cox-2 and PGE2 Protection of colonic structures	[178]
Black raspberries (BRBs)	Male F-344 rats	32	4–5 weeks old	5% whole BRB powder, 0.2% BRB anthocyanins, or 2.25% of the residue fraction	6 weeks	↑ *Anaerostipes, Ruminococcus, Coprobacillus, and* ↓ *Acetivibrio, Anaerotruncus* spp. by 5% whole BRB ↑ *A. muciniphila* by the whole BRB and the residue	[211]
Polyphenol-rich diet	Clinical trial	51	≥60 years old	3 daily portions (1391 mg/day of dietary polyphenols)	8 weeks	Improved intestinal permeability ↑ *Butyrate* ↑ *Roseburia* ↑ *Butyricicoccus* ↑ *Faecalibacterium* ↑ *Butyricicoccus* ↓ *Methanobrevibacter*	[199]
Green tea liquid	Randomized clinical trial	12	27–46 years old	400 mL/day	14 days	↑ α-Diversity; changed microbial composition; ↑ Relative abundance of (family) *Lachnospiraceae* and *Bifidobactericeae*; ↑ Bifidobacterium and SCFA-producing genus *Roseburia, Feacalibacterium, Eubacterium, Blautia, Coprococcus,* and *Dorea*; ↑ Predicted microbial genes for LPS biosynthesis, glutathione metabolism, and glycosaminoglycan degradation	[212]
De-alcoholized red wine	Randomized, crossover, controlled study	10	48 ± 2 years old	733.02 ± 23.61 mg gallic acid equivalent/day	20 days	↑ *Fusobacteria, Bacteroidetes, Enterococcus, Blautia coccoides–Eubacterium rectale, Bifidobacterium*, and *Eggerthella lenta*. ↓ Cholesterol and C-reactive protein	[213]
